# *Tent5a* modulates muscle fiber formation in adolescent idiopathic scoliosis via maintenance of myogenin expression

**DOI:** 10.1111/cpr.13183

**Published:** 2022-02-09

**Authors:** Ming Luo, Huiliang Yang, Diwei Wu, Xuanhe You, Shishu Huang, Yueming Song

**Affiliations:** ^1^ Department of Orthopedic Surgery and Orthopedic Research Institute West China Hospital Sichuan University Chengdu China; ^2^ Department of Orthopedics Zhongnan Hospital Wuhan University Wuhan China

**Keywords:** adolescent idiopathic scoliosis, myoblast differentiation, myogenin, paravertebral muscle asymmetry, *Tent5a*

## Abstract

**Objective:**

Paravertebral muscle asymmetry may be involved in the pathogenesis of adolescent idiopathic scoliosis (AIS), and the Tent5a protein was recently identified as a novel active noncanonical poly(A) polymerase. We, therefore, explored the function of the AIS susceptibility gene *Tent5a* in myoblasts.

**Materials and methods:**

RNA‐seq of AIS paravertebral muscle was performed, and the molecular differences in paravertebral muscle were investigated. Twenty‐four AIS susceptibility genes were screened, and differential expression of *Tent5a* in paravertebral muscles was confirmed with qPCR and Western blot. After the knockdown of *Tent5a*, the functional effects of *Tent5a* on C2C12 cell proliferation, migration, and apoptosis were detected by Cell Counting Kit‐8 assay, wound‐healing assay, and TUNEL assay, respectively. Myogenic differentiation markers were tested with immunofluorescence and qPCR in vitro, and muscle fiber formation was compared in vivo.

**Results:**

The AIS susceptibility gene *Tent5a* was differentially expressed in AIS paravertebral muscles. *Tent5a* knockdown inhibited the proliferation and migration of C2C12 cells and inhibited the maturation of type I muscle fibers in vitro and in vivo. Mechanistically, the expression of myogenin was decreased along with the suppression of Tent5a.

**Conclusions:**

*Tent5a* plays an important role in the proliferation and migration of myoblasts, and it regulates muscle fiber maturation by maintaining the stability of myogenin. *Tent5a* may be involved in the pathogenesis of AIS by regulating the formation of muscle fiber type I.

## Graphical abstract

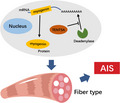

## 1 INTRODUCTION

Adolescent idiopathic scoliosis (AIS) is characterized as a puberty‐onset spinal deformity that affects millions of children worldwide.^1^ Although great progress has been made in the study of AIS over the last 20 years, its pathogenesis is far from fully understood due to its genetic complexity.^2^ Spinal fusion is recommended for AIS patients with Cobb angles exceeding 45°, but the risks and health economics related to surgical treatments are thorny issues.^3^ Clarifying the pathogenesis of AIS is of great significance for its early diagnosis, prevention, and treatment.

The etiology of AIS is multifactorial, including abnormal bone metabolism, endocrine hormone dyscrasia, or neuromuscular system imbalance,^2^ and the genetic basis cannot be ignored in the pathogenesis of AIS.^4^ A functional variant of AIS, Lbx‐As1, was identified in a genome‐wide association study based on 4,317 Chinese AIS patients.^5^ Many susceptibility genes related to skeletal muscle development, such as *Lbx1*, *Pax3*, and *Tbx1*, have been reported in AIS.^6^, ^7^, ^8^ Recently, a large number of studies have reported paravertebral muscle asymmetry based on imaging changes, histological characteristics, and electrophysiological differences,^9^, ^10^, ^11^, ^12^ which indicates that paravertebral muscle asymmetry may be involved in the pathogenesis of AIS.

Recently, 20 loci significantly associated with AIS have been reported from 79,211 Japanese individuals, and *Tent5a* was one of the susceptibility genes for AIS.^13^ *Tent5a* was recently identified as a novel active noncanonical poly(A) polymerase.^14^ Poly(A) length is highly regulated in the nucleus and cytoplasm, and poly(A) tails help regulate gene expression and probably play a critical role in cell differentiation.^15^, ^16^ Tent5a has previously been found to be associated with a variety of human diseases, including retinitis pigmentosa, skeletal disorders, Alzheimer's disease, and obesity.^17^, ^18^, ^19^ However, the function of the *Tent5a* gene in muscle development is still unclear.

Based on the asymmetry of paravertebral muscle in AIS, we explored the function of the AIS susceptibility gene *Tent5a* in myoblasts, as well as the formation of muscle fibers. We revealed that *Tent5a* plays an important role in the proliferation and migration of myoblasts and regulates muscle fiber maturation by maintaining the stability of myogenin. These findings shed new light on the etiology of AIS and provide new therapeutic opportunities for AIS patients to correct muscle asymmetry with nonoperative management.

## 2 MATERIALS AND METHODS

### 2.1 Multifidus specimens of AIS patients

**FIGURE 1:**
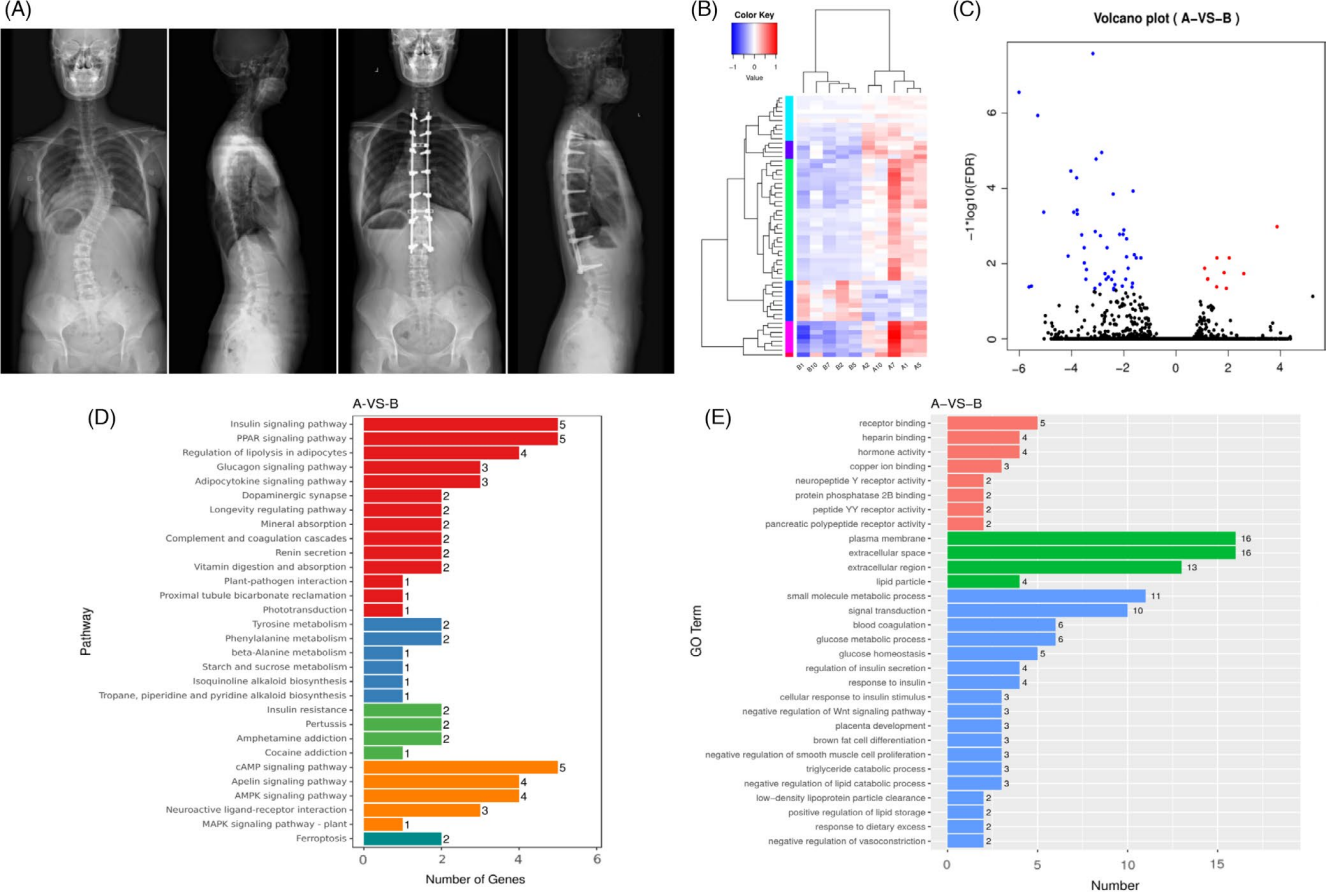
RNA sequencing of AIS paravertebral muscles and differential expression analysis. (A) Representative radiograph of AIS patients who underwent spinal corrective surgery. Muscle tissues were obtained from the multifidus at the apical region of concavity and convexity during surgery. (B) Clustergram of differential expression genes. (C) Volcano Plot of differential expression genes. (D) KEGG pathway of differential expression genes. (E) GO term of differential expression genes

This study was approved by the institutional research ethics board (HX‐2019‐607). Twenty female AIS patients with a right thoracic curve who underwent spinal corrective surgery between the ages of 12 and 18 were included in this study (Figure 1A). In addition, we collected muscle samples from five teenagers who underwent spinal surgery due to spinal cord trauma as the normal control. Detailed information for curve type, age, gender, and Cobb angle of the AIS patients is presented in Table S1. Muscle tissues (~1.0 × 1.0 × 0.5 cm) were obtained from the multifidus at the apical region of concavity and convexity before tissue cautery. Specimens were divided into two parts. One part was frozen in precooled isopentane for immunofluorescence staining, and the other part was flash‐frozen in liquid nitrogen for RNA and protein testing.

### 2.2 RNA‐seq and differential expression analysis

After total RNA was extracted and quantified, library preparation of each sample was constructed according to the manufacturer's protocol with 1 μg total RNA. After isolation of the poly(A) mRNA, mRNA fragmentation and priming were performed using NEBNext First Strand Synthesis Reaction Buffer and NEBNext Random Primers. Then, libraries with different indices were multiplexed and loaded on an Illumina HiSeq instrument according to the manufacturer's instructions (Illumina).

Quality control, mapping, and expression analysis were performed, and the Padj of genes was set to <0.05 to detect differentially expressed genes. GOSeq (v1.34.1) was used to identify Gene Ontology terms that annotate a list of enriched genes with a significant Padj less than 0.05. In addition, topGO was used to plot DAG. We used scripts in‐house to enrich significantly differentially expressed genes in KEGG pathways.

### 2.3 Expression analysis of AIS susceptibility genes

In this study, 24 susceptibility genes reported in East Asian AIS patients according to two large sample size genome‐wide association studies were selected.^5^, ^13^ Zhu et al.^5^ reported five susceptibility genes (*Lbx1*‐*As1*, *Pax3*, *Epha4*, *Bcl2*, and *Ajap1*) with a sample of 4,317 Chinese AIS patients and 6,016 controls. The other 19 susceptibility genes (*Mtmr11*, *Arf1*, *Tbx1*, *Csmd1*, *Kif24*, *Bckdhb*, *Tent5a*, *Creb5*, *Nt5Dc1*, *Uncx*, *Plxna2*, *Agmo*, *Meox2*, *Fto*, *Lbx1*, *Bnc2*, *Abo*, *Pax1*, and *Cdh13*) were identified from a meta‐analysis of three genome‐wide association studies consisting of 79,211 Japanese individuals.^13^ Detailed information on the 24 susceptibility genes is presented in Table S2.

### 2.4 C2C12 cell culture and lentiviral transfection

C2C12 cells were acquired from the Chinese National Collection of Authenticated Cell Cultures (No. SCSP‐505). C2C12 cells were cultured in growth medium (DMEM with 10% fetal bovine serum, Gibco), and myogenic differentiation was induced with differentiation medium (DMEM with 2% horse serum, HyClone) when C2C12 cells grew to 90% confluence.

The knockdown and control lentiviruses were purchased from Wuhan BrainVTA Co., Ltd. The density of C2C12 cells was approximately 80% after inoculation for 24 h, the growth medium was replaced, and concentrated lentivirus was added (MOI = 100). Auxiliary infection reagents were also added, and the growth medium was replaced the next day. Puromycin was used to select Tent5a‐inhibited C2C12 cells. EGFP fluorescence was detected with fluorescence microscopy 48 h after transfection, and qPCR and Western blotting were used to verify the knockdown effectiveness.

### 2.5 Effects of Tent5a knockdown on C2C12 cells

C2C12 cells at 70% confluence were selected and prepared into a single‐cell suspension. To detect the effect of Tent5a knockdown on myoblast viability, C2C12 cells were inoculated in 96‐well plates, and 2000 cells in 100 µl of growth medium were seeded in each well. After incubation for 24 h, the Cell Counting Kit‐8 assay (YZ‐CK04, Solarbio) was performed according to the manufacturer's protocol. The wound‐healing assay was used to quantify cell migration. Cells were inoculated with a 6‐well plate and scratched when the cell density increased close to 100%. The observation time points of the wound‐healing assay included 0, 24, and 48 h. To detect the effect of Tent5a knockdown on myoblast apoptosis, C2C12 cell apoptosis was induced by H_2_O_2,_ and a TUNEL assay (A113, Vazyme) was conducted according to the manufacturer's protocol. After culturing in differentiation medium for 5 days, C2C12 cells were fused and formed mature myotubes. Immunofluorescence staining and qPCR were used to detect the effect of Tent5a knockdown on myoblast differentiation.

### 2.6 Primary cultures and cell transplantation

Satellite cells were isolated by a method described by Watanabe et al.^20^ Briefly, the quadriceps femoris muscles of 4‐week‐old C57BL/6 mice (Dossy Experimental Animals Co., Ltd.) were cut off, and nonmuscular tissues, including fat, fascia, nerves, blood vessels, and tendons, were carefully removed using an asana microscope. The muscle was repeatedly pruned to approximately 1 mm^3^ in size with ophthalmic scissors and digested with 0.1% type I collagenase (C8140, Solarbio) for 1 h. After enzymatic digestion, filtration, and resuspension, satellite cells were incubated in high serum growth medium. Satellite cells were further purified by the differential adhesion method and verified by Pax7 staining (Pax7, DSHB). P3‐5 myoblasts were selected for lentivirus transfection, and the knockdown efficiency of Tent5a expression was confirmed with qPCR and Western blot.

One day before cell transplantation, 4‐week‐old C57BL/6 mice were selected to create a muscle injury model.^21^ Briefly, 50 µl of cardiotoxin (10 µM) was multipoint injected into the tibialis anterior muscle using a 31G insulin syringe. Then, myoblasts transfected with knockdown or control lentivirus were prepared, and 50 µl of single‐cell suspension within 5 × 10^5^ transfected myoblasts was multipoint injected into the tibial anterior muscle.^22^ Four weeks after myoblast transplantation, the tibialis anterior muscles were collected for subsequent experiments.

### 2.7 Immunofluorescence staining

The thickness of the frozen section for AIS samples was 8 µm. After fixation with 4% paraformaldehyde and then blocking with 5% BSA (Biofroxx) in 0.025% PBS‐Tween 100 (Biofroxx), we incubated the slices with primary antibodies against MHC I (1:20, BA‐D5, DSHB) and MHC IIa (1:20, SC‐71, DSHB) overnight at 4°C. The secondary antibodies (1:200, Yeasen) were incubated at room temperature and protected from light for 1 h. Muscle samples of mouse tibialis anterior were processed in the same manner as described above.

For satellite cells and C2C12 cells, 4% paraformaldehyde and 0.5% Triton^®^X‐100 (Biofroxx) were used to incubate cells successively. After blocking, they were stained with the following primary antibodies: Pax7 (1:5, Pax7, DSHB), MHC (1:20, MF 20, DSHB), Myh7 (1:100, sc‐53090, Santa Cruz), myogenin (1:100, sc‐12732, Santa Cruz), and MyoD (1:100, sc‐377460, Santa Cruz). The nuclei were stained with DAPI for 5 min. We collected the images using a fluorescence microscope. ImageJ software (Version 1.52 V) was used to analyze the percentage of myosin heavy chain‐, Myh7‐, myogenin‐, and MyoD‐positive cells.

### 2.8 qPCR and Western blot

After the muscle samples were pulverized using a mortar and pestle in liquid nitrogen or the cells were washed with PBS, RNAiso (108‐95‐2, Takara) was added, and total RNA was isolated with Qiagen RNAeasy Mini Kits (1062832, Qiagen) per the manufacturer's guidelines. The PrimeScript™ RT reagent kit with a gDNA eraser was used to produce cDNA. PowerUp™ SYBR™ green master mix (A25742, Thermo Fisher Scientific) was added to detect the expression of related genes, and the gene‐specific primers are listed in Table S3 and Table S4. Quantitative real‐time PCRs were performed with an ABI QuantStudio 3 machine (Thermo Fisher Scientific).

RIPA lysis solution with 1% PMSF was used to extract total protein. Protein quantification was performed using the BCA method. Gel electrophoresis and membrane transfer were performed according to standard processes. After nonspecific antigen blocking with 5% BSA, primary antibodies including Tent5a (1:200, A12765, ABclonal), MHC (1:50, MF20, DSHB), MHC I (1:50, BA‐D5, DSHB), myogenin (1:500, sc‐12732, Santa Cruz), and Gapdh (1:5000) were used to incubate the polyvinylidene fluoride transfer membrane overnight. Images were captured with the Chemidoctm Imaging System. The relative expression of Tent5a, MHC, and MHC I was calculated according to the gray values using ImageJ software (Version 1.52 V).

### 2.9 Statistical analysis

Data were analyzed and plotted using GraphPad Prism 8.4.3 (GraphPad Software Inc.), and all quantitative data are presented as the mean ± SD. Statistical analyses were carried out using *t*‐tests or rank‐sum tests when comparing continuous variables and the chi‐square test or Fisher's exact test for dichotomous variables. Statistical tests were chosen based on sample size and normality of distribution. A *p* value of < 0.05 was considered statistically significant.

## 3 RESULTS

### 3.1 RNA sequencing of AIS paravertebral muscles

To investigate the molecular differences of the paravertebral muscle in AIS, RNA sequencing of convex and concave paravertebral muscles was performed (Figure 1B). The results showed that the expression of 10 genes was upregulated on the concavity side, and 48 genes were downregulated (Figure 1C). KEGG analysis showed that the differentially expressed genes were enriched mainly in the insulin signaling pathway, PPAR signaling pathway, and cAMP signaling pathway (Figure 1D). GO analysis showed that the differentially expressed genes were enriched mainly in extracellular space, glucose metabolic process, and glucose homeostasis (Figure 1E).

### 3.2 Differential expression of Tent5a in AIS paravertebral muscles

**FIGURE 2:**
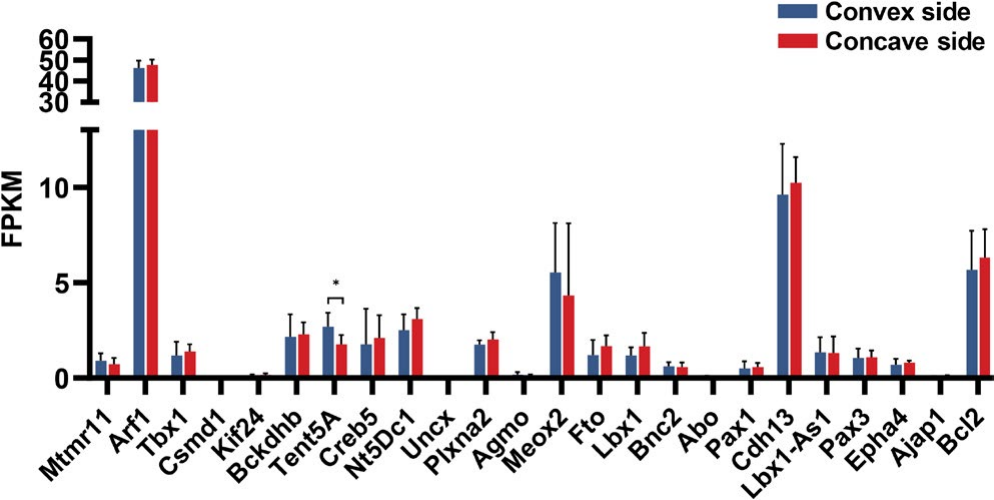
Expression of AIS susceptibility genes in paravertebral muscles. The expression of 24 AIS susceptibility genes was compared between convex and concave sides in paravertebral muscles, and differential expression of Tent5a was found. Each bar represents mean ± SD. Paired sample *t*‐test. *p < 0.05

**FIGURE 3:**
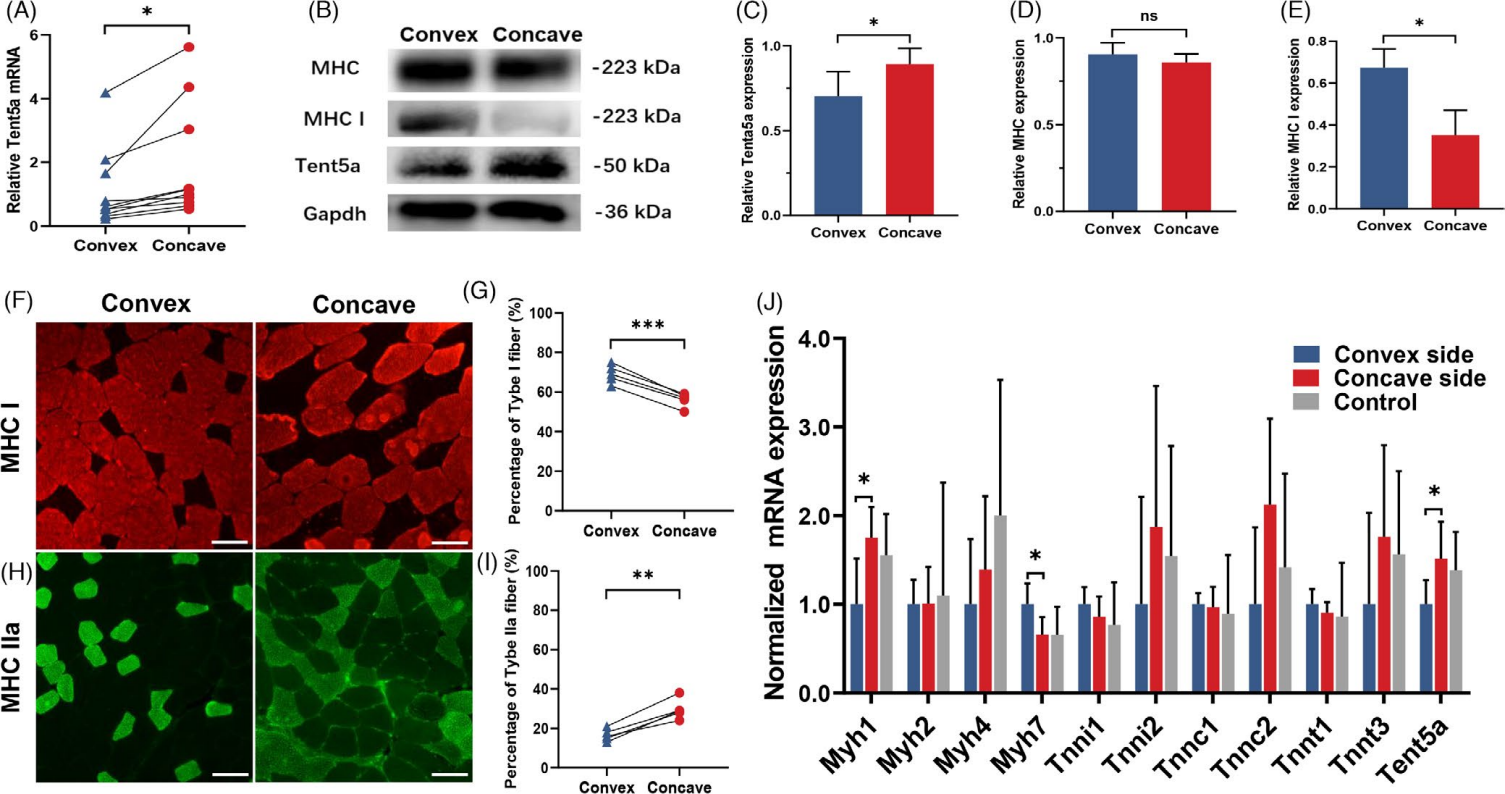
Asymmetry of fiber type and Tent5a expression in AIS paravertebral muscles. (A) The differential expression of Tent5a was verified with qPCR in 10 pairs of paravertebral muscles from AIS patients. Paired sample *t*‐test. *p < 0.05. (B–E) Representative Western blots showing asymmetric expression of Tent5a and MHC I in paravertebral muscles. Each bar represents mean ± SD. Paired sample *t*‐test. *p < 0.05. (F and G) Representative immunofluorescence showing a significantly lower proportion of type I muscle fibers on the concave side. Paired sample *t*‐test. ***p < 0.001. (H and I) Representative immunofluorescence showing a significantly higher proportion of type IIa muscle fibers on the concave side. Paired sample *t*‐test. **p < 0.01. The scale bar represents 50 µm. (J) The relative mRNA expression of myofiber‐related genes among the convex side, concave side, and control muscles. One‐way ANOVA test. *p < 0.05

We screened for the expression of previously reported AIS susceptibility genes from RNA sequencing data, including *Mtmr11*, *Arf1*, *Tbx1*, *Csmd1*, *Kif24*, *Bckdhb*, *Tent5a*, *Creb5*, *Nt5Dc1*, *Uncx*, *Plxna2*, *Agmo*, *Meox2*, *Fto*, *Lbx1*, *Bnc2*, *Abo*, *Pax1*, *Cdh13*, *Lbx1*‐*As1*, *Pax3*, *Epha4*, *Ajap1*, and *Bcl2*.^5^, ^13^ Only the *Tent5a* gene showed differential expression in the two‐sided paravertebral muscles of AIS patients (Figure 2). The differential reads for Tent5a were also confirmed with the use of the IGV tool (Figure S1). We selected another 10 pairs of paravertebral muscles from AIS patients and verified the differential expression of Tent5a with qPCR (Figure 3A) and Western blot (Figure 3B,C).

### 3.3 Asymmetry of fiber type in AIS

Compared with the convex side, a significantly lower proportion of type I muscle fibers was found on the concave side (Figure 3F,G), but the proportion of type IIA muscle fibers was higher (Figure 3H,I). In addition, the expression of MHC and MHC I proteins in the paravertebral muscles of AIS patients was detected by Western blot. There was no significant difference in the relative expression of MHC between the concavity and convex sides (Figure 3B,D), and the MHC I protein on the concave side of the paravertebral muscle was significantly lower than the MHC I protein on the convex side (Figure 3B,E). These results support the asymmetry of fiber type in AIS. In addition, the relative mRNA expressions of myofiber‐related genes were compared among the convex side, concave side, and control muscles. The mRNA expressions in the concave side of AIS, but not the convex side, were closer to the normal control, including Myh1, Myh7, Tnni1, Tnni2, Tnnt3, and Tent5a (Figure 3J).

### 3.4 Tent5a knockdown inhibited the proliferation and migration of C2C12 cells

**FIGURE 4:**
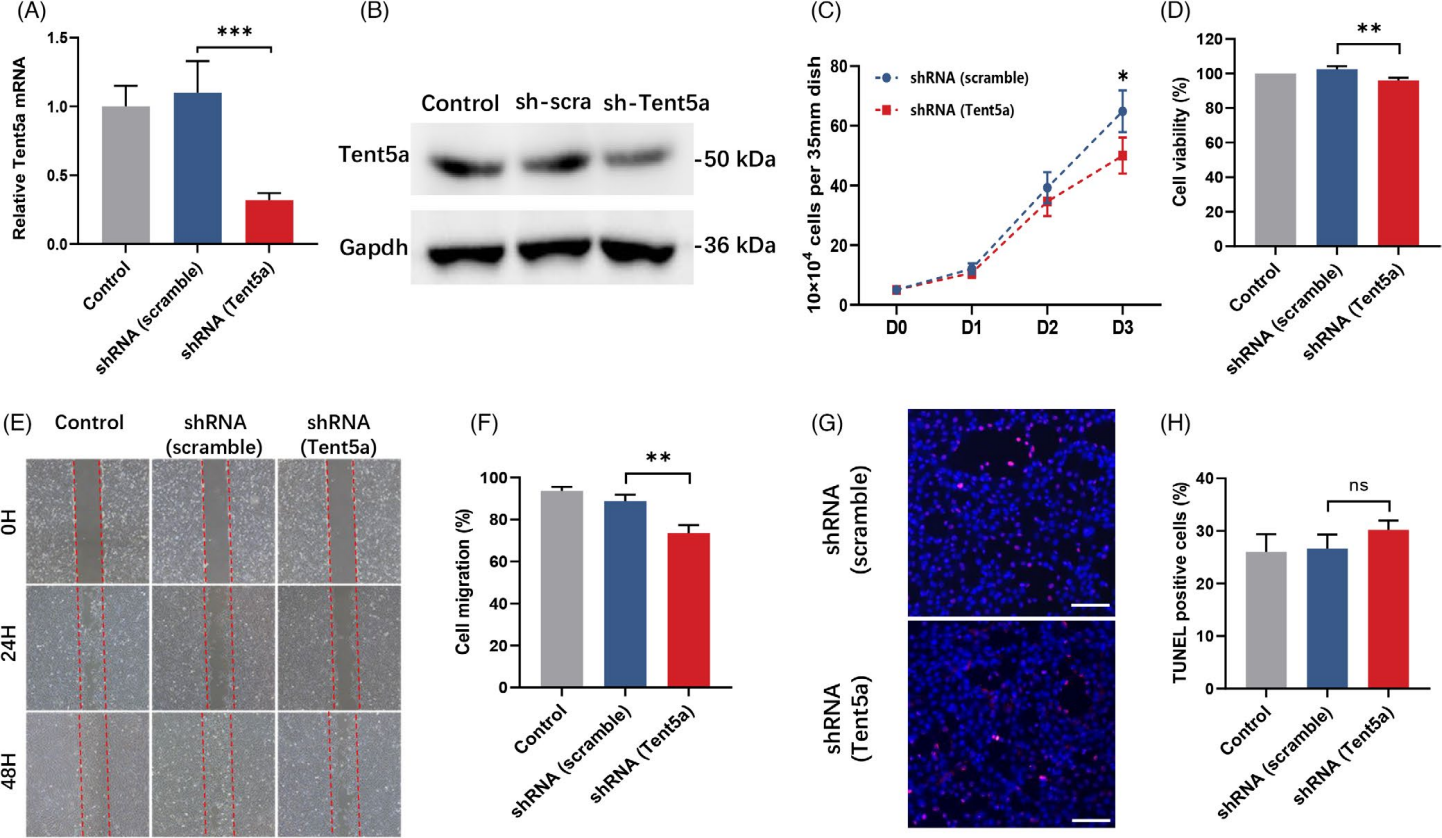
Tent5a knockdown inhibited the proliferation and migration of C2C12 cells. (A and B) qPCR and Western blots were conducted to verify *Tent5a* knockdown in C2C12 cells. Each bar represents mean ± SD. One‐way ANOVA with multiple comparison test. ***p < 0.001. (C and D) Cell counting and Cell Counting Kit‐8 assay showing lower proliferation viability in the Tent5a knockdown group. Each bar represents mean ± SD. One‐way ANOVA with multiple comparison test. *p < 0.05, **p < 0.01. (E and F) Representative wound‐healing assay showing lower migration viability in the Tent5a knockdown group. Each bar represents mean ± SD. One‐way ANOVA with multiple comparison test. **p < 0.01. (G and H) Representative TUNEL assay showing a comparable percentage of apoptosis among groups. Each bar represents mean ± SD. One‐way ANOVA with multiple comparison test. The scale bar represents 200 µm

To confirm the effect of Tent5a on myoblasts, we produced Tent5a knockdown C2C12 cells by transfection with lentivirus. Cell counting showed that the cell number of the Tent5a knockdown group was significantly lower than the cell number of the *Tent5a* knockdown group of the negative control group on Day 3 (Figure 4C). In addition, the Cell Counting Kit‐8 assay showed lower cell viability in the Tent5a knockdown group (Figure 4D).

The effect of Tent5a knockdown on the migration ability of C2C12 cells was detected with a wound‐healing assay. After 48 h of culture, the migration area in the Tent5a knockdown group was significantly lower than the migration area in the *Tent5a* knockdown group in the control group (Figure 4E,F). A TUNEL assay was used to detect the effect of Tent5a knockdown on the apoptosis of C2C12 cells. The percentage of apoptosis was comparable between the two groups. (Figure 4G,H). These results suggest that Tent5a knockdown inhibited the proliferation and migration of C2C12 cells.

### 3.5 Tent5a knockdown inhibited myogenic differentiation of C2C12 cells

**FIGURE 5:**
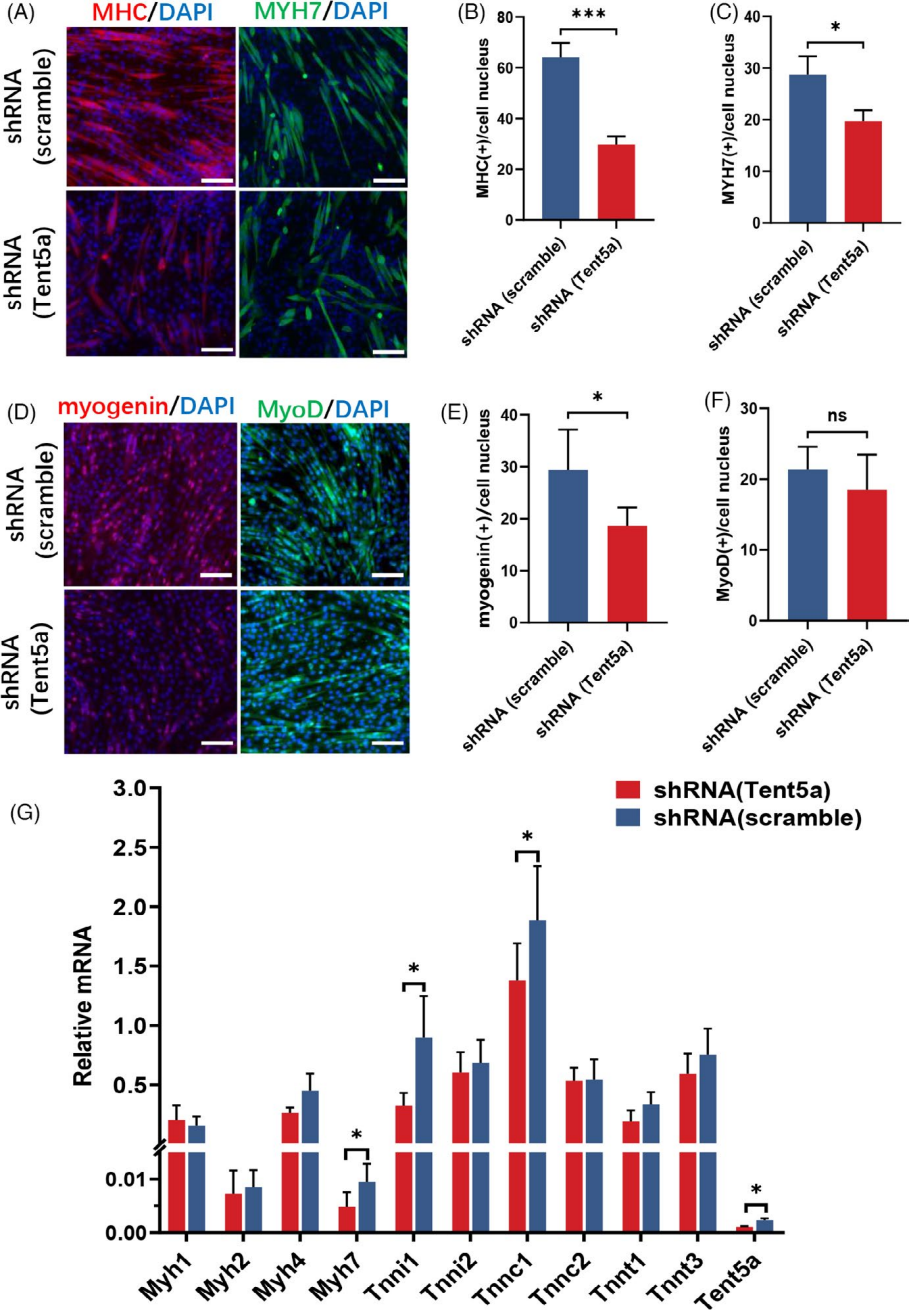
Tent5a knockdown inhibited myogenic differentiation of C2C12 cells. (A–C) Representative immunofluorescence showing a lower intensity and density of MHC and MYH7, as well as the percentage of MHC (+) nuclei and MYH7 (+) nuclei in the Tent5a knockdown group. Each bar represents mean ± SD. Unpaired *t*‐test. *p < 0.05, ***p < 0.001. (D–F) Representative immunofluorescence showing a lower percentage of myogenin (+) nuclei in the *Tent5a* knockdown group. Each bar represents mean ± SD. Unpaired *t*‐test. *p < 0.05. (G) qPCR showing decreased mRNA expression of Myh7, Tnni1, and Tnnc1 in the Tent5a knockdown group. Each bar represents mean ± SD. Unpaired *t*‐test. *p < 0.05. The scale bar represents 100 µm

After 5 days of myogenic differentiation, the expression of the myogenic protein was detected with immunofluorescence staining. Compared with the negative control group, the intensity and density of immunofluorescence for MHC were significantly lower in the Tent5a knockdown group (Figure 5A). The percentage of MHC (+) nuclei was also decreased in the Tent5a knockdown group (Figure 5B). Myogenin, a myogenic transcription factor essential for myogenic differentiation, was decreased in the Tent5a knockdown group (Figure 5D–F). These results suggested that Tent5a knockdown inhibited the myogenic differentiation of C2C12 cells.

Intriguingly, we found that the deregulation of Tent5a led to a significant decrease in the mRNA expression of Myh7, Tnni1, and Tnnc1 (Figure 5G). Furthermore, the percentage of Myh7 (+) nuclei was also decreased in the Tent5a knockdown group (Figure 5C). These results suggested that Tent5a knockdown inhibited mainly myogenic differentiation and especially impaired the maturation of genes related to type I muscle fibers.

### 3.6 Tent5a knockdown inhibited the maturation of type I muscle fibers in vivo

**FIGURE 6:**
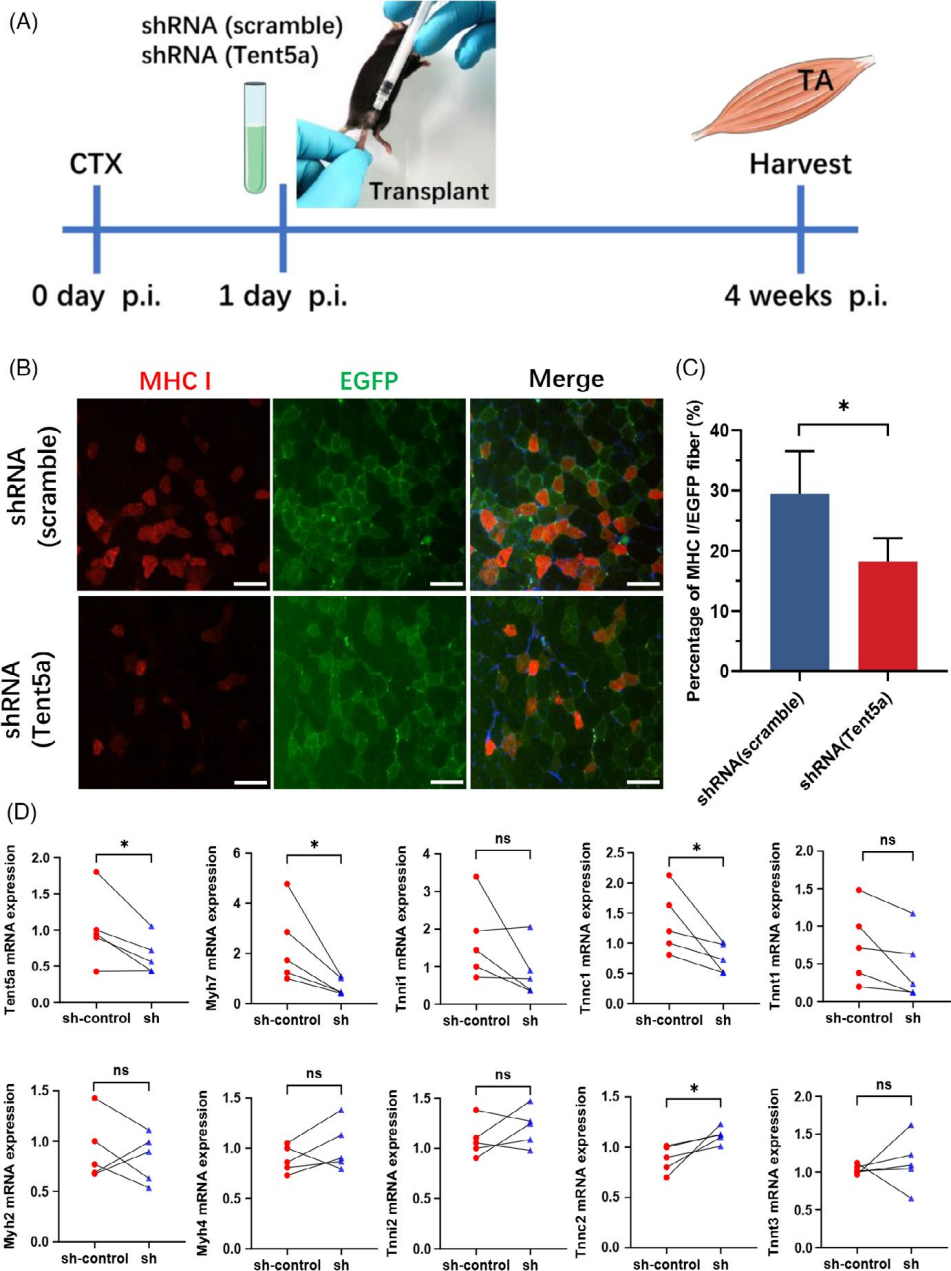
Tent5a knockdown inhibited the maturation of type I muscle fibers in vivo. (A) Paradigm showing the operation process in vivo. (B and C) Representative immunofluorescence showing a lower percentage of MHC I/EGFP muscle fibers in the Tent5a knockdown group. Each bar represents mean ± SD. Paired *t*‐test. *p < 0.05. (D) qPCR showing decreased mRNA expression of Myh7 and Tnnc1 in the Tent5a knockdown group. Each bar represents mean ± SD. Paired *t*‐test. *p < 0.05. The scale bar represents 100 µm

To investigate the effect of Tent5a on myogenic differentiation in vivo, myoblasts were labeled with EGFP using lentiviral transfection and transplanted into the tibial anterior muscle of mice (Figure 6A). EGFP‐positive muscle fibers represented the myogenic differentiation of transplanted cells. Four weeks later, we found that the percentage of MHC I/EGFP muscle fibers in the Tent5a knockdown group was significantly lower than the percentage of MHC I/EGFP muscle fibers in the control group (Figure 6B,C). In the Tent5a knockdown group, the relative mRNA expression of Myh7 and Tnnc1 was also significantly decreased, while the relative expression of the *Tnnc2* gene was increased (Figure 6D). These results confirmed that Tent5a knockdown mainly inhibited the maturation of type I muscle fibers in vivo.

### 3.7 Regulatory effects of Tent5a on myogenic regulatory factors

**FIGURE 7:**
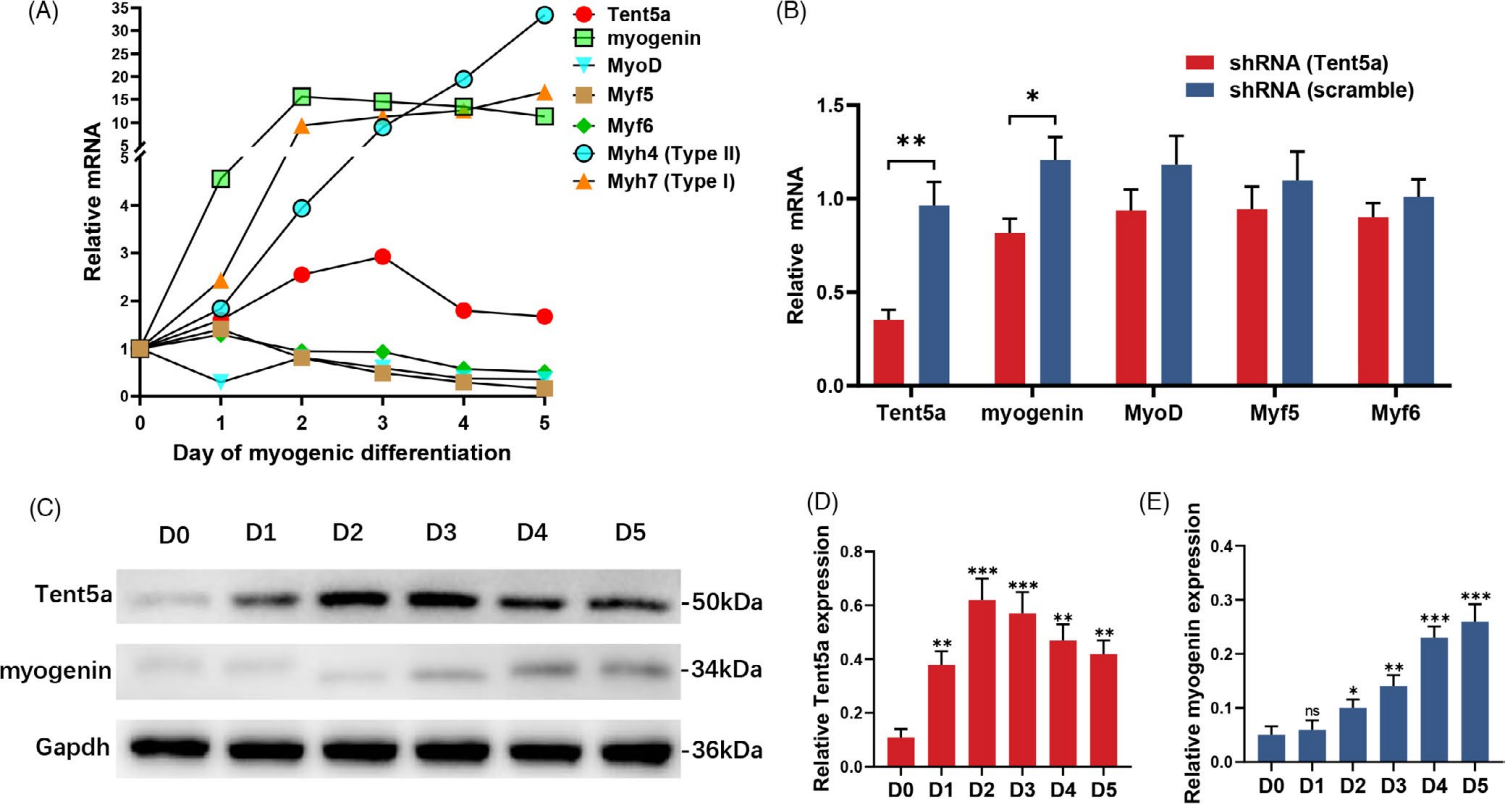
Regulatory effects of Tent5a on myogenic regulatory factors. (A) The dynamic expression of Tent5a and myogenic regulatory factors (myogenin, MyoD, Myf5, and Myf6) during myogenic differentiation. (B) qPCR showing decreased mRNA expression of myogenin in the Tent5a knockdown group. Each bar represents mean ± SD. Unpaired *t*‐test. *p < 0.05, **p < 0.01. (C–E) Representative Western blots showing dynamic expression of Tent5a and myogenin during myogenic differentiation. Each bar represents mean ± SD. Unpaired *t*‐test. *p < 0.05, **p < 0.01, ***p < 0.001

We detected the mRNA expression of Tent5a, myogenic regulatory factors (myogenin, MyoD, Myf5, and Myf6), and myogenic‐related genes (Myh4 and Myh7). The relative expression of Tent5a was upregulated from Day 0 to Day 3 and downregulated gradually from Day 4 to Day 5 (Figure 7A). The relative expression of myogenin was significantly upregulated on the first day and maintained at a high level on Day 5 (Figure 7A). The Western blot results showed that the relative expression of Tent5a was upregulated from Day 0 to Day 2 and then maintained at high expression levels during myoblast differentiation (Figure 7C,D), and the relative expression of myogenin was increased progressively from Day 0 to Day 5 (Figure 7C,E).

To investigate the regulatory effects of Tent5a on myogenic regulatory factors, we conducted Tent5a knockdown experiments, and the mRNA expression levels of myogenin, MyoD, Myf5, and Myf6 were detected on Day 3 of myogenic differentiation. The relative expression of myogenin was significantly downregulated in the Tent5a knockdown group (Figure 7B). The previous results also showed decreased myogenin (+) nuclei in the Tent5a knockdown group (Figure 5D–F). These results suggested that Tent5a knockdown mainly restrained the expression of myogenin.

## 4 DISCUSSION

**FIGURE 8:**
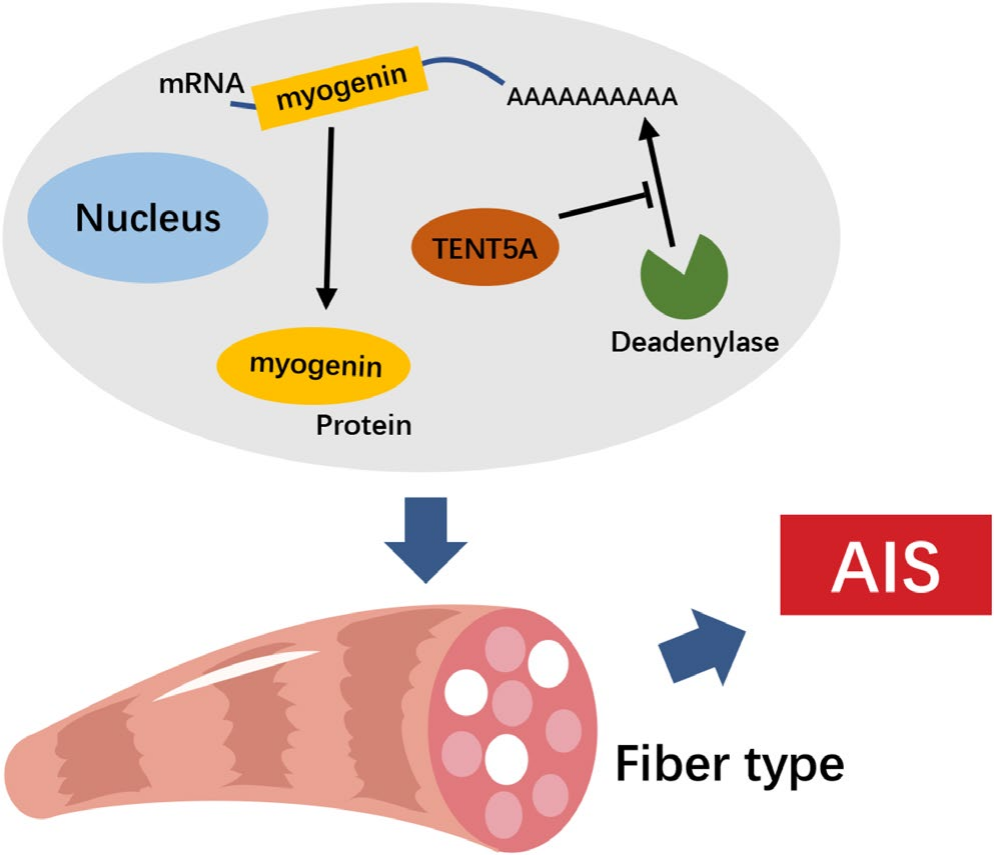
Schematic drawing of Tent5a modulates muscle fiber formation in adolescent idiopathic scoliosis. The poly(A) polymerase Tent5a promotes myogenic differentiation by maintaining the mRNA stability of myogenin, and it is involved in the pathogenesis of AIS by modulating muscle fiber formation

Because of the genetic complexity of AIS, its pathogenesis has not been clarified. In contrast to congenital scoliosis, no significant vertebral dysplasia is observed in the early stages of AIS.^23^, ^24^ Recently, an increasing number of studies have speculated that paravertebral muscle asymmetry may be involved in the pathogenesis of AIS.^25^, ^26^ Here, we investigated the role of the susceptibility gene *Tent5a* in the pathogenesis of AIS, and we found that Tent5a knockdown inhibited the proliferation and migration of C2C12 cells and inhibited the maturation of type I muscle fibers in vitro and in vivo. In addition, the *Tent5a* gene might maintain myogenic differentiation by promoting the expression of myogenin. Together, these results indicate that the AIS susceptibility gene Tent5a may be involved in the pathogenesis of AIS by modulating muscle fiber formation via maintenance of myogenin expression (Figure 8).

Although paravertebral muscle asymmetry has been reported in AIS, the molecular differences in the paravertebral muscle of AIS are unclear. We conducted RNA‐seq to compare the convex and concave paravertebral muscles. Fifty‐eight genes were differentially expressed in AIS paraspinal muscle, and these genes were mainly enriched in the insulin signaling pathway, PPAR signaling pathway, and cAMP signaling pathway. The insulin signaling pathway is crucial for the regulation of glucose levels and energy metabolism, and its downstream molecules play important roles in glycogen synthesis, protein synthesis, glucose transport, and cell apoptosis.^27^, ^28^ Glucose metabolism and insulin sensitivity are essential for muscle contraction, and previous evidence has shown that muscle disorders could cause abnormal lipid deposition.^29^ Recently, FR030200‐Tent5a signaling has been reported to participate in the insulin resistance of the vascular injury.^30^ In addition, *Tent5a* has been validated as a trans‐regulator for leptin, and *Tent5a* knockdown increased insulin‐stimulated leptin secretion from the adipocytes.^19^ PPAR belongs to the ligand‐activated receptor family of nuclear hormone receptors. One of its subtypes, PPARα, is involved in lipid metabolism and regulates the expression of many myogenic genes.^31^, ^32^, ^33^ Similarly, scholars recently revealed the differential expression of H19 and ADIPOQ in the paravertebral muscles of AIS patients, and they speculated that transcriptome differences in AIS patients might be the reason for the structural and functional imbalance of paravertebral muscles.^34^

Investigating the function of AIS susceptibility genes could help us to further understand the pathogenesis of AIS. Recently, several AIS susceptibility genes, such as *Lbx1* and *Pax3*, have been identified to be associated with skeletal muscle function.^5^, ^8^, ^35^, ^36^ We screened 24 AIS susceptibility genes and verified the differential expression of Tent5a in the paravertebral muscle. Furthermore, we knocked down the expression of Tent5a and inhibited the proliferation and migration of C2C12 cells. During the development of somatic segments, sarcomeres originate mainly from the directional migration of precursor cells in the dermomyotome and then fuse into mature muscle fibers.^37^, ^38^, ^39^, ^40^ Sufficient quantities of myoblasts and their normal migration function are needed to ensure precise directional migration during somatic segmental development.^41^, ^42^ Otherwise, impaired myoblasts might lead to an asymmetric distribution of epaxial muscle and eventually result in the morphological and functional imbalance of paravertebral muscles. Therefore, Tent5a might interfere with skeletal muscle development by impairing the proliferation and migration function of myoblasts.

The myoblasts could fuse and transform into fast‐twitch (type I) and slow‐twitch (type IIa, type IIb, and type IIx) skeletal muscle fibers in vivo. The function of skeletal muscle depends largely on different proportions of muscle fibers, and its delicate combination is essential for complex body movement and postural behavior.^43^, ^44^ In AIS patients, we found different proportions of type I and type IIa muscle fibers between the concave and convex sides of the paravertebral muscles, which supported the asymmetry of paravertebral muscle in AIS. In addition, we found that Tent5a knockdown inhibited myogenic differentiation and especially impaired the maturation of genes related to type I muscle fibers. The composition of muscle fibers is regulated by many factors. Exercise could promote an increased proportion of type I muscle fibers and enable the transition from fast to slow muscles.^45^ Tbx1 was reported to regulate the muscle fiber type and oxidative metabolism in the myotube and stimulate the differentiation of myoblasts.^46^ Taken together, these results indicate that *Tent5a* may be involved in the pathogenesis of AIS by disturbing muscle fiber formation.

Tent5a has recently been identified as an active atypical poly(A) polymerase.^14^ Poly(A) polymerase plays a highly modulatory role in the nucleus and cytoplasm, and different lengths of poly(A) tails could influence the stability, transport, and translation of mRNAs.^15^, ^16^ Recently, Tent5a was identified as a trans‐regulator of leptin through proteomic analysis.^19^ Patients identified with mutation of *Tent5a* were associated with severe osteogenesis imperfecta, and skeletal dysplasia was found in the mouse with *Tent5a* mutation.^47^, ^48^ In addition, Tent5a was expressed in hematopoietic system cells and played an important role in heme‐induced hemoglobinization.^49^ Deletion of the *Tent5a* gene led to abnormal formation of eye and body color in Xenopus.^50^ In this study, we first reported the function of Tent5a in myoblasts and myogenic differentiation in vitro and in vivo.

As the core member of myogenic regulator factors, myogenin regulates the fate of myoblasts and culminates in the formation of mature muscle fibers.^51^ In this study, we found that Tent5a knockdown mainly restrains the expression of myogenin. Previous studies have shown that myogenin processed myoblast fusion and determined the number and size of muscle fibers.^52^ MyoD and myogenin are an overlapping group of myogenic regulatory factors that maintain the expression of each other in a feed‐forward cycle. Rather than MyoD, late maturation depends mainly on myogenin, and MyoD cannot compensate for the loss of myogenin in vivo.^53^ In addition to controlling the maturation of skeletal muscle, myogenin also affected the location and distribution of resident satellite cells, which demonstrated a multilevel contribution of myogenin to muscle homeostasis throughout life.^54^ Therefore, the poly(A) polymerase Tent5a might play an important role in maintaining the mRNA stability of myogenin. The risks and health economics related to spinal correction in AIS are thorny issues, and Tent5a might be a new regulatory target for muscle development. Our findings provide new therapeutic opportunities to correct muscle asymmetry with nonoperative management.

Some limitations could not be ignored in this study. Firstly, the mechanism for *Tent5a* in maintaining the mRNA stability of myogenin deserves further study. Secondly, it is not an easy task to collect back muscle samples from normal teenagers, only five control muscle samples have been collected in this study, and a larger sample size control study is needed to verify the muscle difference between AIS and normal teenagers. Finally, changes in mRNA and protein expression for Tent5a and myofiber‐related genes after spinal fusion for AIS are still unclear.

## 5 CONCLUSIONS

The poly(A) polymerase Tent5a plays an important role in the proliferation and migration of myoblasts, and it might regulate muscle fiber maturation by maintaining the stability of myogenin. As the susceptibility gene of AIS, *Tent5a* may be involved in the pathogenesis of AIS by regulating the formation of muscle fiber type I. These findings provide new therapeutic opportunities to correct muscle asymmetry for AIS with nonoperative management.

## CONFLICT OF INTEREST

The authors have declared that no competing interest exists.

## AUTHOR CONTRIBUTIONS

YMS and SSH designed the project. ML, HLY, DWW, and XHY collected and analyzed the data. ML and HLY wrote the manuscript. All authors reviewed the manuscript.

## Supporting information

Figure S1

Table S1‐S4

## Data Availability

I confirm that my article contains a Data Availability Statement even if no data is available (list of sample statements) unless my article type does not require one (e.g., Editorials, Corrections, Book Reviews, etc.).

## References

[1] Altaf F , Gibson A , Dannawi Z , Noordeen H . Adolescent idiopathic scoliosis. BMJ. 2013;346:f2508. doi:10.1136/bmj.f2508 23633006

[2] Cheng JC , Castelein RM , Chu WC , et al. Adolescent idiopathic scoliosis. Nat Rev Dis Primers. 2015;1(1):15030. doi:10.1038/nrdp.2015.30 27188385

[3] Weinstein SL , Dolan LA , Cheng JC , Danielsson A , Morcuende JA . Adolescent idiopathic scoliosis. Lancet. 2008;371(9623):1527‐1537. doi:10.1016/s0140-6736(08)60658-3 18456103

[4] Kou I , Takahashi Y , Johnson TA , et al. Genetic variants in GPR126 are associated with adolescent idiopathic scoliosis. Nat Genet. 2013;45(6):676‐679. doi:10.1038/ng.2639 23666238

[5] Zhu Z , Tang NL , Xu L , et al. Genome‐wide association study identifies new susceptibility loci for adolescent idiopathic scoliosis in Chinese girls. Nat Commun. 2015;6(1):8355. doi:10.1038/ncomms9355 26394188 PMC4595747

[6] Li Y , Wu Z , Xu L , et al. Genetic variant of TBX1 gene is functionally associated with adolescent idiopathic scoliosis in the Chinese population. Spine. 2021;46(1):17‐21. doi:10.1097/brs.0000000000003700 32947497

[7] Xu L , Sheng F , Xia C , et al. Genetic variant of PAX1 gene is functionally associated with adolescent idiopathic scoliosis in the Chinese population. Spine. 2018;43(7):492‐496. doi:10.1097/brs.0000000000002475 29095406

[8] Zhu Z , Xu L , Leung‐Sang Tang N , et al. Genome‐wide association study identifies novel susceptible loci and highlights Wnt/beta‐catenin pathway in the development of adolescent idiopathic scoliosis. Hum Mol Genet. 2017;26(8):1577‐1583. doi:10.1093/hmg/ddx045 28334814

[9] Watanabe K , Ohashi M , Hirano T , et al. The influence of lumbar muscle volume on curve progression after skeletal maturity in patients with adolescent idiopathic scoliosis: a long‐term follow‐up study. Spine Deform. 2018;6(6):691‐698.e1. doi:10.1016/j.jspd.2018.04.003 30348345

[10] Wright J , Herbert MA , Velazquez R , Bobechko WP . Morphologic and histochemical characteristics of skeletal muscle after long‐term intramuscular electrical stimulation. Spine. 1992;17(7):767‐770. doi:10.1097/00007632-199207000-00007 1502640

[11] Farahpour N , Younesian H , Bahrpeyma F . Electromyographic activity of erector spinae and external oblique muscles during trunk lateral bending and axial rotation in patients with adolescent idiopathic scoliosis and healthy subjects. Clin Biomech (Bristol, Avon). 2015;30(5):411‐417. doi:10.1016/j.clinbiomech.2015.03.018 25846325

[12] Farahpour N , Ghasemi S , Allard P , Saba MS . Electromyographic responses of erector spinae and lower limb's muscles to dynamic postural perturbations in patients with adolescent idiopathic scoliosis. J Electromyogr Kinesiol. 2014;24(5):645‐651. doi:10.1016/j.jelekin.2014.05.014 25008019

[13] Kou I , Otomo N , Takeda K , et al. Genome‐wide association study identifies 14 previously unreported susceptibility loci for adolescent idiopathic scoliosis in Japanese. Nat Commun. 2019;10(1):3685. doi:10.1038/s41467-019-11596-w 31417091 PMC6695451

[14] Kuchta K , Muszewska A , Knizewski L , et al. FAM46 proteins are novel eukaryotic non‐canonical poly(A) polymerases. Nucleic Acids Res. 2016;44(8):3534‐3548. doi:10.1093/nar/gkw222 27060136 PMC4857005

[15] Subtelny AO , Eichhorn SW , Chen GR , Sive H , Bartel DP . Poly(A)‐tail profiling reveals an embryonic switch in translational control. Nature. 2014;508(7494):66‐71. doi:10.1038/nature13007 24476825 PMC4086860

[16] Zhao T , Huan Q , Sun J , et al. Impact of poly(A)‐tail G‐content on Arabidopsis PAB binding and their role in enhancing translational efficiency. Genome Biol. 2019;20(1):189. doi:10.1186/s13059-019-1799-8 31481099 PMC6724284

[17] Schott JM , Crutch SJ , Carrasquillo MM , et al. Genetic risk factors for the posterior cortical atrophy variant of Alzheimer's disease. Alzheimers Dement. 2016;12(8):862‐871. doi:10.1016/j.jalz.2016.01.010 26993346 PMC4982482

[18] Gewartowska O , Aranaz‐Novaliches G , Krawczyk PS , et al. Cytoplasmic polyadenylation by TENT5A is required for proper bone formation. Cell Rep. 2021;35(3):109015. doi:10.1016/j.celrep.2021.109015 33882302

[19] Carayol J , Chabert C , Di Cara A , et al. Protein quantitative trait locus study in obesity during weight‐loss identifies a leptin regulator. Nat Commun. 2017;8(1):2084. doi:10.1038/s41467-017-02182-z 29234017 PMC5727191

[20] Watanabe S , Kondo S , Hayasaka M , Hanaoka K . Functional analysis of homeodomain‐containing transcription factor Lbx1 in satellite cells of mouse skeletal muscle. J Cell Sci. 2007;120(Pt 23):4178‐4187. doi:10.1242/jcs.011668 18003701

[21] Guardiola O , Andolfi G , Tirone M , Iavarone F , Brunelli S , Minchiotti G . Induction of acute skeletal muscle regeneration by cardiotoxin injection. J vis Exp. 2017;119:54515. doi:10.3791/54515 PMC5407614 28117768

[22] Motohashi N , Asakura Y , Asakura A . Isolation, culture, and transplantation of muscle satellite cells. J vis Exp. 2014;(86):e50846. doi:10.3791/50846 PMC4131689 24747722

[23] Pérez‐Machado G , Berenguer‐Pascual E , Bovea‐Marco M , et al. From genetics to epigenetics to unravel the etiology of adolescent idiopathic scoliosis. Bone. 2020;140:115563. doi:10.1016/j.bone.2020.115563 32768685

[24] Ramo BA , McClung A , Jo CH , Sanders JO , Yaszay B , Oetgen ME . Effect of etiology, radiographic severity, and comorbidities on baseline parent‐reported health measures for children with early‐onset scoliosis. J Bone Joint Surg Am. 2021;103(9):803‐811. doi:10.2106/jbjs.20.00819 33439608

[25] Brzoska E , Kalkowski L , Kowalski K , et al. Muscular contribution to adolescent idiopathic scoliosis from the perspective of stem cell‐based regenerative medicine. Stem Cells Dev. 2019;28(16):1059‐1077. doi:10.1089/scd.2019.0073 31170887

[26] Schmid S , Burkhart KA , Allaire BT , et al. Spinal compressive forces in adolescent idiopathic scoliosis with and without carrying loads: a musculoskeletal modeling study. Front Bioeng Biotechnol. 2020;8:159. doi:10.3389/fbioe.2020.00159 32195239 PMC7062648

[27] Kubota T , Kubota N , Kadowaki T . Imbalanced insulin actions in obesity and type 2 diabetes: key mouse models of insulin signaling pathway. Cell Metab. 2017;25(4):797‐810. doi:10.1016/j.cmet.2017.03.004 28380373

[28] Akhtar A , Sah SP . Insulin signaling pathway and related molecules: role in neurodegeneration and Alzheimer's disease. Neurochem Int. 2020;135:104707. doi:10.1016/j.neuint.2020.104707 32092326

[29] Fan L , Sweet DR , Prosdocimo DA , et al. Muscle Krüppel‐like factor 15 regulates lipid flux and systemic metabolic homeostasis. J Clin Invest. 2021;131(4):e139496. doi:10.1172/jci139496 PMC7880311 33586679

[30] Liu S , Zheng F , Cai Y , Zhang W , Dun Y . Effect of long‐term exercise training on lncRNAs expression in the vascular injury of insulin resistance. Cardiovasc Transl Res. 2018;11(6):459‐469. doi:10.1007/s12265-018-9830-0 30302742

[31] Cheng HS , Tan WR , Low ZS , Marvalim C , Lee JYH , Tan NS . Exploration and development of PPAR modulators in health and disease: an update of clinical evidence. Int J Mol Sci. 2019;20(20):5055. doi:10.3390/ijms20205055 PMC6834327 31614690

[32] Mirza AZ , Althagafi II , Shamshad H . Role of PPAR receptor in different diseases and their ligands: physiological importance and clinical implications. Eur J Med Chem. 2019;166:502‐513. doi:10.1016/j.ejmech.2019.01.067 30739829

[33] Nakamura MT , Yudell BE , Loor JJ . Regulation of energy metabolism by long‐chain fatty acids. Prog Lipid Res. 2014;53:124‐144. doi:10.1016/j.plipres.2013.12.001 24362249

[34] Jiang H , Yang F , Lin T , et al. Asymmetric expression of H19 and ADIPOQ in concave/convex paravertebral muscles is associated with severe adolescent idiopathic scoliosis. Mol Med. 2018;24(1):48. doi:10.1186/s10020-018-0049-y 30241458 PMC6145194

[35] Jennings W , Hou M , Perterson D , et al. Paraspinal muscle Ladybird Home Box 1 (LBX1) in adolescent idiopathic scoliosis: a cross‐sectional study. Spine J. 2019;19(12):1911‐1916. doi:10.1016/j.spinee.2019.06.014 31202838

[36] Qin X , He Z , Yin R , Qiu Y , Zhu Z . Abnormal paravertebral muscles development is associated with abnormal expression of PAX3 in adolescent idiopathic scoliosis. Eur Spine J. 2020;29(4):737‐743. doi:10.1007/s00586-019-06217-5 31832874

[37] Endo T . Molecular mechanisms of skeletal muscle development, regeneration, and osteogenic conversion. Bone. 2015;80:2‐13. doi:10.1016/j.bone.2015.02.028 26453493

[38] McColl J , Mok GF , Lippert AH , Ponjavic A , Muresan L , Münsterberg A . 4D imaging reveals stage dependent random and directed cell motion during somite morphogenesis. Sci Rep. 2018;8(1):12644. doi:10.1038/s41598-018-31014-3 30139994 PMC6107556

[39] Nakajima T , Shibata M , Nishio M , et al. Modeling human somite development and fibrodysplasia ossificans progressiva with induced pluripotent stem cells. Development. 2018;145(16):dev165431. doi:10.1242/dev.165431 30139810 PMC6124548

[40] Musumeci G , Castrogiovanni P , Coleman R , et al. Somitogenesis: from somite to skeletal muscle. Acta Histochem. 2015;117(4–5):313‐328. doi:10.1016/j.acthis.2015.02.011 25850375

[41] Chal J , Pourquié O . Making muscle: skeletal myogenesis in vivo and in vitro. Development. 2017;144(12):2104‐2122. doi:10.1242/dev.151035 28634270

[42] Yvernogeau L , Auda‐Boucher G , Fontaine‐Perus J . Limb bud colonization by somite‐derived angioblasts is a crucial step for myoblast emigration. Development. 2012;139(2):277‐287. doi:10.1242/dev.067678 22129828

[43] Matsuoka Y , Inoue A . Controlled differentiation of myoblast cells into fast and slow muscle fibers. Cell Tissue Res. 2008;332(1):123‐132. doi:10.1007/s00441-008-0582-z 18278513

[44] Braun T , Gautel M . Transcriptional mechanisms regulating skeletal muscle differentiation, growth and homeostasis. Nat Rev Mol Cell Biol. 2011;12(6):349‐361. doi:10.1038/nrm3118 21602905

[45] Moreillon M , Conde Alonso S , Broskey NT , et al. Hybrid fiber alterations in exercising seniors suggest contribution to fast‐to‐slow muscle fiber shift. J Cachexia Sarcopenia Muscle. 2019;10(3):687‐695. doi:10.1002/jcsm.12410 30907516 PMC6596392

[46] Motohashi N , Uezumi A , Asakura A , et al. Tbx1 regulates inherited metabolic and myogenic abilities of progenitor cells derived from slow‐ and fast‐type muscle. Cell Death Differ. 2019;26(6):1024‐1036. doi:10.1038/s41418-018-0186-4 30154444 PMC6748120

[47] Doyard M , Bacrot S , Huber C , et al. FAM46A mutations are responsible for autosomal recessive osteogenesis imperfecta. J Med Genet. 2018;55(4):278‐284. doi:10.1136/jmedgenet-2017-104999 29358272

[48] Diener S , Bayer S , Sabrautzki S , et al. Exome sequencing identifies a nonsense mutation in Fam46a associated with bone abnormalities in a new mouse model for skeletal dysplasia. Mamm Genome. 2016;27(3–4):111‐121. doi:10.1007/s00335-016-9619-x 26803617

[49] Lin H‐H , Lo Y‐L , Wang W‐C , Huang K‐Y , I K‐Y , Chang G‐W . Overexpression of FAM46A, a non‐canonical poly(A) polymerase, promotes hemin‐induced hemoglobinization in K562 cells. Front Cell Dev Biol. 2020;8:414. doi:10.3389/fcell.2020.00414 32528962 PMC7264091

[50] Watanabe T , Yamamoto T , Tsukano K , Hirano S , Horikawa A , Michiue T . Fam46a regulates BMP‐dependent pre‐placodal ectoderm differentiation in Xenopus. Development. 2018;145(20):dev166710. doi:10.1242/dev.166710 30291163

[51] Hernández‐Hernández O , Ávila‐Avilés RD , Hernández‐Hernández JM . Chromatin landscape during skeletal muscle differentiation. Frontiers in Genetics. 2020;11:578712. doi:10.3389/fgene.2020.578712 33193700 PMC7530293

[52] Ganassi M , Badodi S , Ortuste Quiroga HP , Zammit PS , Hinits Y , Hughes SM . Myogenin promotes myocyte fusion to balance fibre number and size. Nat Commun. 2018;9(1):4232. doi:10.1038/s41467-018-06583-6 30315160 PMC6185967

[53] Adhikari A , Kim W , Davie J . Myogenin is required for assembly of the transcription machinery on muscle genes during skeletal muscle differentiation. PLoS One. 2021;16(1):e0245618. doi:10.1371/journal.pone.0245618 33465133 PMC7815108

[54] Ganassi M , Badodi S , Wanders K , Zammit PS , Hughes SM . Myogenin is an essential regulator of adult myofibre growth and muscle stem cell homeostasis. eLife. 2020;9:e60445. doi:10.7554/eLife.60445 33001028 PMC7599067

