# Supplementary figures and images for: Tent5a modulates muscle fiber formation in adolescent idiopathic scoliosis via maintenance of myogenin expression
Source: Cell Prolif. 2022 Feb 9;55(3):e13183. doi: 10.1111/cpr.13183 (PMC8891553; doi:10.1111/cpr.13183)

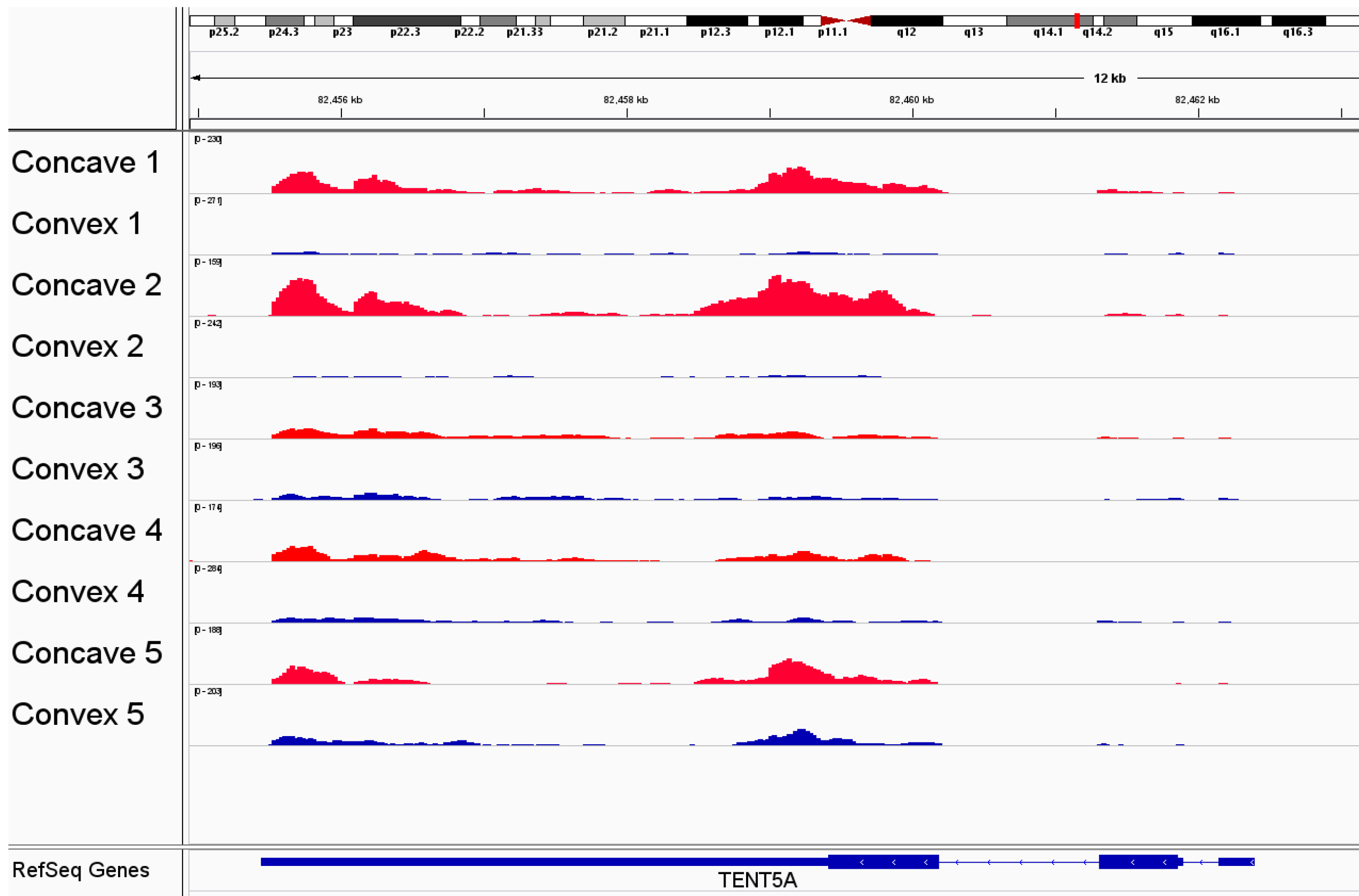

Supplement: Supplementary file 1 — Figure S1 [file CPR-55-e13183-s001.tif]
