# Supplementary material for: Tent5a modulates muscle fiber formation in adolescent idiopathic scoliosis via maintenance of myogenin expression
Source: Cell Prolif. 2022 Feb 9;55(3):e13183. doi: 10.1111/cpr.13183 (PMC8891553; doi:10.1111/cpr.13183)
Supplement: Supplementary file 2 — Table S1‐S4 [file CPR-55-e13183-s002.docx]

**Supplementary Material**

| **Parameters** | **Mean±SD** |
| --- | --- |
| Lenke classification (type 1/2/3/4/5/6) | 13/5/2/0/0/0 |
| Age (year) | 14.65±1.57 |
| Sex (female) | 20 |
| Cobb angle of major curve (degree) | 54.20±5.38 |

**Supplement Table 1.** Detailed information of the included AIS patients. SD, standard deviation.

| **Susceptibility locus** | **Gene in or near region** | **RA** | **OR (95% CI)** |
| --- | --- | --- | --- |
| rs11205303 | MTMR11 | C | 1.17（1.11-1.23） |
| rs12029076 | ARF1 | G | 1.18（1.12-1.24） |
| rs1978060 | TBX1 | G | 1.16（1.11-1.22） |
| rs11787412 | CSMD1 | A | 1.14（1.09-1.18） |
| rs188915802 | KIF24 | T | 1.66（1.41-1.96） |
| rs658839 | BCKDHB/TENT5A | G | 1.14（1.09-1.19） |
| rs160335 | CREB5 | G | 1.13（1.08-1.18） |
| rs482012 | NT5DC1 | T | 1.14（1.09-1.19） |
| rs11341092 | UNCX | AC | 1.14（1.09-1.19） |
| rs17011903 | PLXNA2 | A | 1.20（1.13-1.28） |
| rs397948882 | AGMO/MEOX2 | A | 1.20（1.12-1.28） |
| rs12149832 | FTO | G | 1.16（1.10-1.22） |
| rs11190870 | LBX1 | T | 1.52（1.46-1.59） |
| rs7028900 | BNC2 | C | 1.20（1.15-1.25） |
| rs144131194 | ABO | - | 1.15（1.11-1.20） |
| rs6047716 | PAX1 | C | 1.15（1.11-1.20） |
| rs2194285 | CDH13 | G | 1.19（1.12-1.27） |
| rs678741 | LBX1-AS1 | A | 1.42 (1.27–1.60) |
| rs13398147 | PAX3/EPHA4 | T | 1.48 (1.29–1.70) |
| rs241215 | AJAP1 | A | 0.75 (0.65–0.85) |
| rs4940576 | BCL2 | T | 1.38 (1.23–1.54) |

**Supplement Table 2.** Susceptibility loci for adolescent idiopathic scoliosis in East Asian. RA risk allele, OR odds ratio, CI confidence interval.

| **Gene** | **Primer** | |
| --- | --- | --- |
| GAPDH, F | GGAGCGAGATCCCTCCAAAAT | |
| GAPDH, R | GGCTGTTGTCATACTTCTCATGG | |
| TENT5A, F | CAACAGTGGCAAAAATGTGG | |
| TENT5A, R | TCCTGGAAATCGCCATAGAC | |
| MYH7, F | CTTTGCTGTTATTGCAGCCATT | |
| MYH7, R | | AGATGCCAACTTTCCTGTTGC |
| MYH1, F | | CCCTACAAGTGGTTGCCAGTG |
| MYH1, R | | CTTCCCTGCGCCAGATTCTC |
| MYH2, F | | AGAAACTTCGCATGGACCTAGA |
| MYH2, R | | CCAAGTGCCTGTTCATCTTCA |
| MYH4, F | | ACAAGGTTCTAAATGCGAGTGC |
| MYH4, R | | TGACCGAATTTGTACTGGGTG |
| TNNI1, F | | TCCGTGGGAAGTTCAAGCG |
| TNNI1, R | | GACTTGGCGGCATCAAACATC |
| TNNC1, F | | TGGTTCGGTGCATGAAGGAC |
| TNNC1, R | | GTCGATGTAGCCATCAGCATT |
| TNNT1, F | | AACGCGAACGTCAGGCTAAGCT |
| TNNT1, R | | CAGGGAGAAACGACCTGGAG |
| TNNI2, F | | ATCTGCGGGGCAAGTTCAAG |
| TNNI2, R | | AGGACTCGGACTCAAACATCT |
| TNNC2, F | | TGGGGACATCAGCGTCAAG |
| TNNC2, R | | CCAAGAACTCCTCGAAGTCGAT |
| TNNT3, F | | AGGAGCTGGTCGCTCTCAA |
| TNNT3, R | | CCTTCTCTGCACGAATCCTCT |

**Supplement Table 3.** The real time quantitative PCR primers for human.

| **Gene** | **Primer** |
| --- | --- |
| GAPDH, F | AGGTCGGTGTGAACGGATTTG |
| GAPDH, R | TGTAGACCATGTAGTTGAGGTCA |
| MYH7, F | GCCAACTATGCTGGAGCTGATGCCC |
| MYH7, R | GGTGCGTGGAGCGCAAGTTTGTCATAAG |
| MYH1, F | GGCAGCAGCAGCTGCGGAAGCAGAGTCTGG |
| MYH1, R | GAGTGCTCCTCAGATTGGTCATTAGC |
| MYH2, F | GGCACAAACTGCTGAAGCAGAGGC |
| MYH2, R | GGTGCTCCTGAGGTTGGTCATCAGC |
| MYH4, F | GAGCTACTGGATGCCAGTGAGCGC |
| MYH4, R | CTGGACGATGTCTTCCATCTCTCC |
| TNNI1, F | TGAAGCCAAATGCCTCCACAACAC |
| TNNI1, R | ACACCTTGTGCTTAGAGCCCAGTA |
| TNNC1, F | AGCTCATGAAGGACGGTGACAAGA |
| TNNC1, R | AACCGTGCAAGACCAGCATCTACT |
| TNNT1, F | TGGATCCACCAGCTGGAATCAGAA |
| TNNT1, R | GCTGATGCGGTTGTAGAGCACATT |
| TNNI2, F | AGCAGCAAGGAGCTGGAAGA |
| TNNI2, R | ATGGCGTCGGCAGACATAC |
| TNNC2, F | CCATCATCGAGGAGGTGGAC |
| TNNC2, R | CTTCCCCTTCGCATCCTCTT |
| TNNT3, F | AACTGGAGACTGACAAATTCGAGT |
| TNNT3, R | GCTGTGCTTCTGGGTTTGGT |
| TENT5A, F | GAGGGCGAAGGGTACTTTGC |
| TENT5A, R | TTGCTCCCAGTTCAGCACATT |
| myogenin, F | GAGACATCCCCCTATTTCTACCA |
| myogenin, R | GCTCAGTCCGCTCATAGCC |
| MYOD, F | CGCCTGAGCAAAGTGAATG |
| MYOD, R | AGACCTTCGATGTAGCGGAT |
| MYF5, F | AAGGCTCCTGTATCCCCTCAC |
| MYF5, R | TGACCTTCTTCAGGCGTCTAC |
| MYF6, F | AGAGGGCTCTCCTTTGTATCC |
| MYF6, F | AGAGGGCTCTCCTTTGTATCC |

**Supplement Table 4.** The real time quantitative PCR primers for mouse.
